# The Prognostic Significance of RIMKLB and Related Immune Infiltrates in Colorectal Cancers

**DOI:** 10.3389/fgene.2022.818994

**Published:** 2022-04-04

**Authors:** Yinghao Cao, Shenghe Deng, Lizhao Yan, Junnan Gu, Fuwei Mao, Yifan Xue, Le Qin, Zhengxing Jiang, Wentai Cai, Changmin Zheng, Xiu Nie, Hongli Liu, Zhuolun Sun, Fumei Shang, Kaixiong Tao, Jiliang Wang, Ke Wu, Bin Zhu, Kailin Cai

**Affiliations:** ^1^ Department of Gastrointestinal Surgery, Union Hospital, Tongji Medical College, Huazhong University of Science and Technology, Wuhan, China; ^2^ Department of Hand Surgery, Union Hospital, Tongji Medical College, Huazhong University of Science and Technology, Wuhan, China; ^3^ College of Life Science and Technology, Huazhong University of Science and Technology, Wuhan, China; ^4^ School of Optical and Electronic Information, Huazhong University of Science and Technology, Wuhan, China; ^5^ Department of Pathology, Union Hospital, Tongji Medical, Huazhong University of Science and Technology, Wuhan, China; ^6^ Cancer Center, Union Hospital, Tongji Medical College, Huazhong University of Science and Technology, Wuhan, China; ^7^ Department of Urology, Third Affiliated Hospital of Sun Yat-sen University, Guangzhou0, China; ^8^ Department of Medical Oncology, Nanyang Central Hospital, Nanyang, China; ^9^ Department of Infectious Diseases, Union Hospital, Tongji Medcial College, Huazhong University of Science and Technology, Wuhan, China

**Keywords:** colorectal cancer, RIMKLB, tumor-infiltrating immune cells, prognosis, biomarker

## Abstract

RimK-like family member B (RIMKLB) is an enzyme that post-translationally modulates ribosomal protein S6, which can affect the development of immune cells. Some studies have suggested its role in tumor progression. However, the relationships among RIMKLB expression, survival outcomes, and tumor-infiltrating immune cells (TIICs) in colorectal cancer (CRC) are still unknown. Therefore, we analyzed RIMKLB expression levels in CRC and normal tissues and investigated the correlations between RIMKLB and TIICs as well as the impact of RIMKLB expression on clinical prognosis in CRC using multiple databases, including the Tumor Immune Estimation Resource (TIMER), Gene Expression Profiling Interactive Analysis (GEPIA), PrognoScan, and UALCAN databases. Enrichment analysis was conducted with the cluster Profiler package in R software to explore the RIMKLB-related biological processes involved in CRC. The RIMKLB expression was significantly decreased in CRC compared to normal tissues, and correlated with histology, stage, lymphatic metastasis, and tumor status (*p* < 0.05). Patients with CRC with high expression of RIMKLB showed poorer overall survival (OS) (HR = 2.5,*p* = 0.00,042), and inferior disease-free survival (DFS) (HR = 1.9,*p* = 0.19) than those with low expression of RIMKLB. TIMER analysis indicated that RIMKLB transcription was closely related with several TIICs, including CD4^+^ and CD8^+^ T cells, B cells, tumor-associated macrophages (TAMs), monocytes, neutrophils, natural killer cells, dendritic cells, and subsets of T cells. Moreover, the expression of RIMKLB showed significant positive correlations with infiltrating levels of PD1 (r = 0.223, *p* = 1.31e-06; r = 0.249, *p* = 1.25e-03), PDL1 (r = 0.223, *p* = 6.03e-07; r = 0.41, *p* = 5.45e-08), and CTLA4 (r = 0.325, *p* = 9.68e-13; r = 0.41, *p* = 5.45e-08) in colon and rectum cancer, respectively. Enrichment analysis showed that the RIMKLB expression was positively related to extracellular matrix and immune inflammation-related pathways. In conclusion, RIMKLB expression is associated with survival outcomes and TIICs levels in patients with CRC, and therefore, might be a potential novel prognostic biomarker that reflects the immune infiltration status.

## Introduction

Colorectal cancer (CRC) is the third most common cancer and the second leading cause of cancer-related death (Siegel et al., 2021). The global incidence of CRC is expected to increase to 2.5 million new cases by 2035, with a steady and declining trend only in highly developed countries (Bray et al., 2018). Furthermore, studies show that CRC is now beginning to develop at a younger age (Dekker et al., 2019; Mauri et al., 2019). In CRC management, metastasis is an important biological feature leading to poor prognosis. Immune-related mechanisms play an important role in digestive tract cancer, especially in CRC (Ganesh et al., 2019; Siegel et al., 2019). In the past decade, a high tumor mutation burden has become a hallmark of immunotherapeutic response in some tumor types due to the success of immunotherapy in achieving long-lasting responses in previously difficult-to-treat solid tumors (Samstein et al., 2019). However, the clinical efficacy of CRC immunotherapy in metastatic CRC is poor, and the popularly used anti-PD-1 and anti-PD-L1 show partial reactions in metastatic CRC and gastric cancer (Galon et al., 2007; Rahma and Hodi, 2019). In these tumors, low tumor mutation load and lack of immune cell infiltration are thought to be mechanisms of immune resistance (Galon et al., 2007). In addition, increasingly more and more studies have found that tumor immune cell infiltration is closely related to the prognosis and efficacy of CRC chemotherapy and immunotherapy (Rahma and Hodi, 2019). Therefore, it is of great significance to elucidate the immunophenotype of CRC-immune interaction, the mechanism of immunotherapeutic resistance, and the identification of new immune-related therapeutic targets.

RIMK is a unique protein that in *Escherichia* coli that acts as an ATP-dependent enzyme that induces oligo-glutamylation of ribosomal protein S6 (S6) after transcription, and bacterial S6 is the target of oligo-glutamylation of ATP-dependent glutamate ligase RIMK (Kino et al., 2011; Pletnev et al., 2019). In *Pseudomonas aeruginosa*, the lack of RIMK can shorten its survival time owing to the functional effect of RIMK on ribosome properties (Grenga et al., 2017). RIMKLB is a mammalian homologous gene of RIMK, it has been cloned in mammals, resulting in β-citrylglutamate (β-CG) or N-acetylaspartylglutamate synthase activity (Collard et al., 2010). Some studies have found that RIMKLB can affect reproductive function of mammals, and in tumor research, RIMKLB may coordinate with DDIT4 function to mediate mTOR inhibition and growth inhibition of tumor cells (Wang et al., 2015; Maekura et al., 2021). Some studies have found that RIMKL modeling can accurately predict the 5-years survival rate of patient with colon cancer patients, suggesting that RIMKL may play a role in tumor progression (Huang et al., 2021). However, the specific role of RIMKLB in CRC is unknown and needs further study.

The tumor microenvironment and tumor-infiltrating immune cells play an important role in CRC tumor progression. The immune components of the tumor microenvironment can regulate tumor progression and are attractive therapeutic targets. A large number of studies have shown that high a infiltration rate of CD8^+^ and CD4^+^ T cells is associated with better prognosis in patients with CRC patients (Tada et al., 2016). Furthermore, high infiltration of dendritic cells (DCs) in tumors has been reported to be associated with more favorable clinical outcomes (Gulubova et al., 2012). Some studies have also shown that extensive infiltration of NK cells in tumors has a good prognostic effect on CRC (Bindea et al., 2013). However, there are no studies have been reported on RIMKLB and the immune microenvironment in CRC, and it remains unknown whether RIMKLB can affect immune cells and tumor microenvironment and promote tumor progression.

Therefore, based on a list of public databases, our study aimed to determine the correlation between RIMKLB expression and tumor-infiltrating immune cells (TIICs) in CRC. Moreover, we also performed subgroup analysis *via* tumor site to determine whether the role of RIMKLB in colon cancer is different from that in rectum cancer.

## Materials and Methods

### UALCAN and Tumor Immune Estimation Resource Database Analysis

The expression level of the RIMKLB gene in various types of cancers was identified in the UALCAN (http://ualcan.path.uab.edu/cgi-bin/ualcan-res-prot.pl) (Chandrashekar et al., 2017) and TIMER database (https://cistrome.shinyapps.io/timer/) (Li et al., 2017). In addition, we focused on an easy-to-use webtool, GEPIA, which is available at http://gepia.cancer-pku.cn/index.html, to study the differential expression of RIMKLB mRNA in CRC tissues and normal tissues (Tang et al., 2017).

### GEPIA and PrognoScan Database Analysis

Using logarithmic rank test, GEPIA was used to generate survival curves, including overall survival (OS) and disease-free survival (DFS), based on gene expression in colon and rectal cancer. The association between RIMKLB expression and OS in CRC was analyzed *via* PrognoScan database (http://www.abren.net/PrognoScan/) (Mizuno et al., 2009), whose data are different from that of The Cancer Genome Atlas (TCGA) database. The threshold was adjusted to a Cox *p*-value < 0.05.

### Tumor Immune Estimation Resource Database

The TIMER database includes 10,897 samples across 32 cancer types based on RNA-Seq expression profiling data from TCGA database. It can test the differential gene expression in tumor tissues, the abundance of TIICs from gene expression profiles, and the statistical correlation between the two genes by the statistical method through gene expression data (Li et al., 2016). Therefore, we analyzed the relationship between RIMKLB expression and TIICs, including CD4^+^ and CD8^+^ T cells, B cells, neutrophils, DCs, and macrophages.

Additionally, the correlation between RIMKLB expression and gene markers of TIICs, including CD8^+^ T cells, T cells (general), B cells, monocytes, tumor-associated macrophages (TAMs), M1 macrophages, M2 macrophages, neutrophils, natural killer (NK) cells, DCs, T-helper 1 (Th1) cells, T-helper 2 (Th2) cells, follicular helper T (Tfh) cells, T-helper 17 (Th17) cells, Tregs, and exhausted T cells, was explored through related modules, which were reported in a previous study (Wu et al., 2020).

### Gene Correlation Identification in GEPIA

The GEPIA database contains the gene expression data from 8,587 normal and 9,736 tumor tissue samples of TCGA and the Genotype-Tissue Expression (GTEx) projects, and can be used to further identify the significantly correlated genes in TIMER (Tang et al., 2017). GEPIA was also used to generate survival curves and determine OS and DFS rates, differential gene expression, and the relationship between two genes. The spearman method was used to determine the correlation coefficient, and a median value of the RIMKLB expression was used as a cutoff to distinguish high expression from low expression.

### Oncogenomics and Mutational Study

We use cBioPortal6 to analyze the impact of the RIMKLB gene in the Colorectal Adenocarcinoma TCGA PanCancer dataset containing 594 samples. Further using the mRNA expression data of the top 25 positively correlated genes to indicate the correlated gene with RIMKLB in CRC. The cancer type summary tab provides a detailed overview of the RIMKLB gene in different subtypes of CRC, i.e., mucinous adenocarcinoma of colon and rectum, colon adenocarcinoma, and rectal adenocarcinoma. It also showed mutations in CRC’s RIMKLB gene and mutations within the associated genome. Different types of mutations associated with the RIMKLB gene in CRC were analyzed using COSMIC-“Catalogue of Somatic Mutations in Cancer.”

### Enrichment Analysis

Patients with CRC were initially divided into high RIMKLB expression and low expression groups. Genes that were differentially expressed between the two groups were screenedto explore the functional role of RIMKLB in CRC with the false discovery rate less than 0.05, and |logFC| ≥ 1 combined with *p* value less than 0.05 were regarded as significant.

### Statistical Analysis

The data were analyzed using the GraphPad Prism (version 6.0) and SPSS (version 21.0). Low and high RIMKLB groups were established based on the median expression of RIMKLB transcription in the separate datasets. Survival curves were generated from the PrognoScan, Kaplan-Meier plots and GEPIA database. The relation of RIMKLB expression and TICSs was evaluated by Spearman’s correlation, and the strength of the correlation was determined using the following guide for the absolute value: 0.00–0.29 (weak), 0.30–0.59 (moderate), 0.60–0.79 (strong), 0.80–1.00 (very strong) (Gao et al., 2017). *p*-values <0.05 were considered statistically significant.

## Result

### The Expression Levels Analysis of RimK-Like Family Member B in Different Types of Human Cancers and Normal Tissues

**FIGURE 1:**
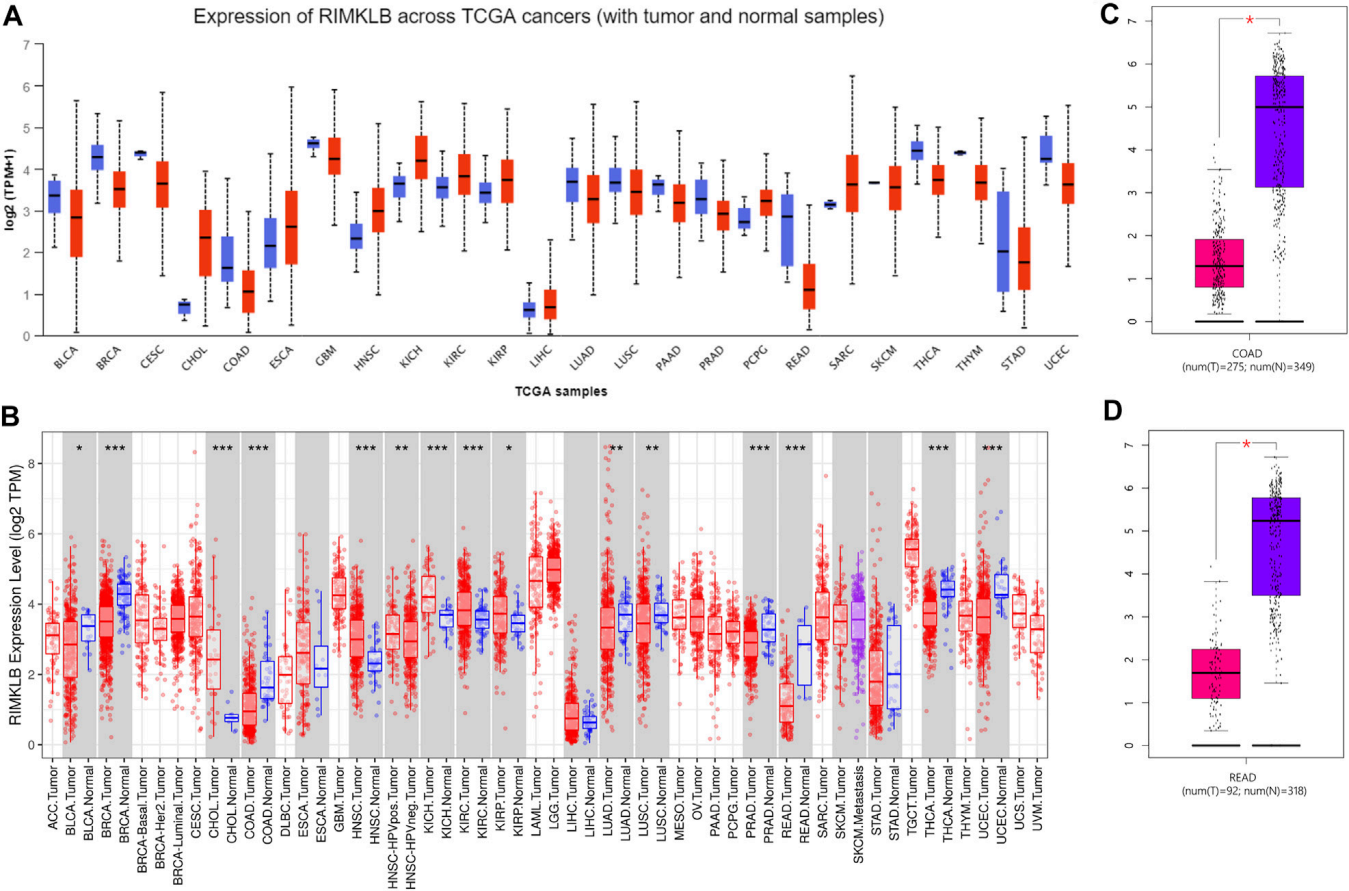
RIMKLB expression levels in different types of human cancers. **(A)** Increased or decreased RIMKLB in data sets of different cancers compared with normal tissues in the UALCAN database; **(B)** Human RIMKLB expression levels in different tumor types from TCGA database were determined by TIMER (*p < 0.05, **p < 0.01, ***p < 0.001); **(C)** Decreased RIMKLB in colon cancer compared with normal tissues; **(D)** Decreased RIMKLB in rectum cancer compared with normal tissues.

To determine the difference expression of RIMKLB between tumor and normal tissue, we used the UALCAN database to analyze the expression levels of RIMKLB in normal tissues of different tumors and multiple cancer types. The result showed that RIMKLB expression was lower in bladder, breast, cervical, cloln, rectum, glioblastoma, lung, pancreatic, prostate, thyroid, thymoma, stomach, uterine corpus endometrial carcinoma, and it was higher in cholangio, esophageal, head and neck, kidney, pheochromocytoma and paraganglioma, sarcomav (Figure 1A). TIMER database was utilized to validate the expression profiles of RIMKLB in pancancer, and RIMKLB mRNA was also lowly expressed in CRC tissues (Figure 1B). The GEPIA database was used to analyze the expression of RIMKLB TPM in colon cancer (Figure 1C) and rectum cancer (Figure 1D). Red represents colon cancer tissue; purple represents normal colon tissue, which is statistically significant (*p* < 0.05).

### Relationship Between RimK-Like Family Member B Expression and Clinicopathological Characteristics of Patients With Colorectal Cancer

**FIGURE 2:**
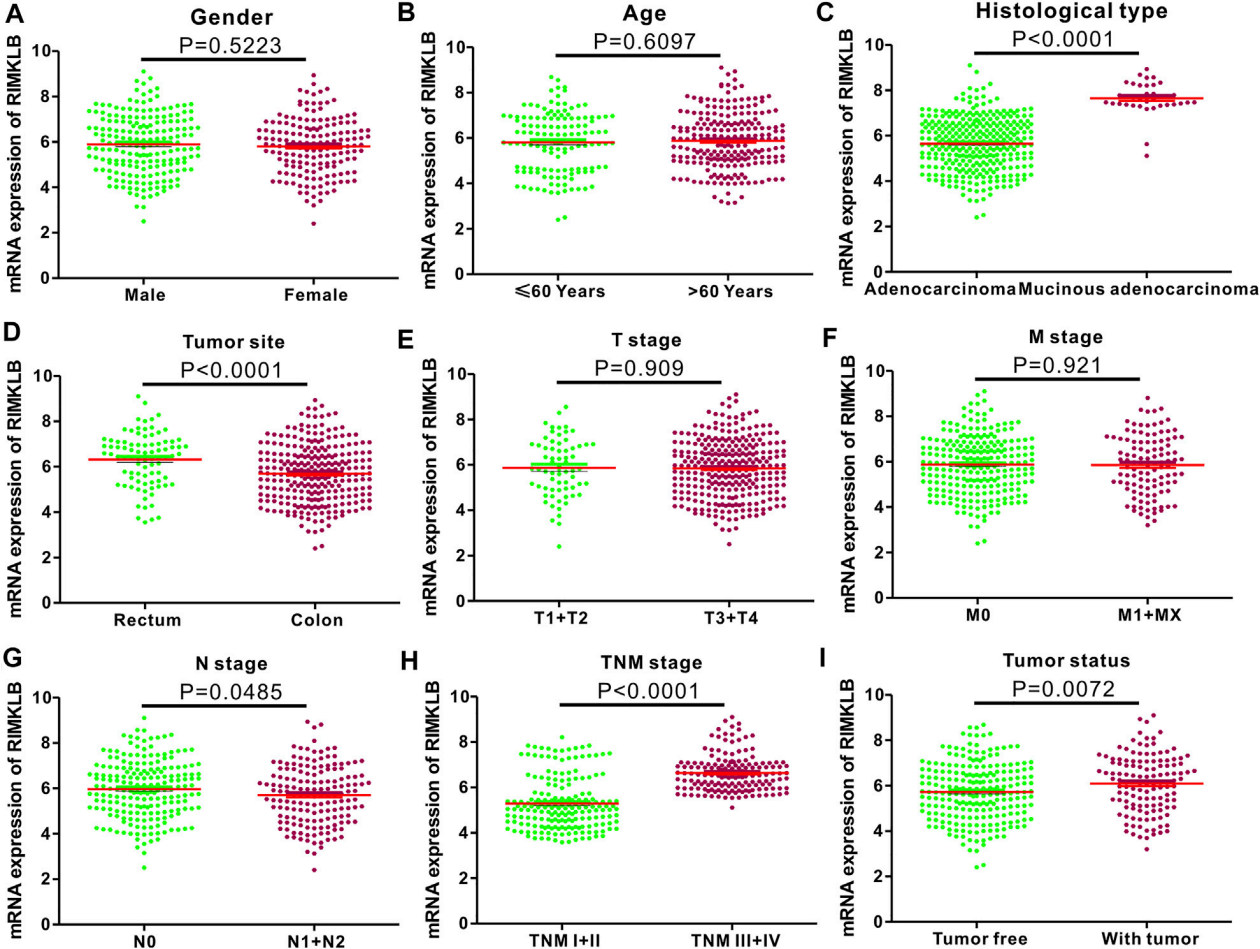
Correlation between RIMKLB mRNA expression and clinical indexes of CRC patients from TCGA database. RIMKLB mRNA expression is stratified with scatter plots using the TCGA dataset by gender **(A)**, gender **(B)**, histological type **(C)**, Tumor site **(D)**, T stage **(E)**, M stage **(F)**, N stage **(G)**, TNM stage **(H)**, and Tumor status **(I)**.

To investigate the relationship between mRNA expression of RIMKLB and clinicopathological features of CRC patients, we analyzed clinical information from CRC samples from the TCGA project. The results (Figure 2) revealed that the mRNA expression of RIMKLB was significantly increased in the mucinous adenocarcinoma (*p* < 0.001), rectum (*p* < 0.001), lymph node stage (N0) (*p* = 0.0485), advanced stages (III/IV) (*p* < 0.001), and with tumor (*p* < 0.001). However, there was no significant correlation between RIMKLB mRNA expression and gender (*p* = 0.5223), age (*p* = 0.6097), advanced tumor (*p* = 0.909) and metastasis status (*p* = 0.921).

### Prognostic Significance of RimK-Like Family Member B Expression in Colorectal CanceC

**FIGURE 3:**
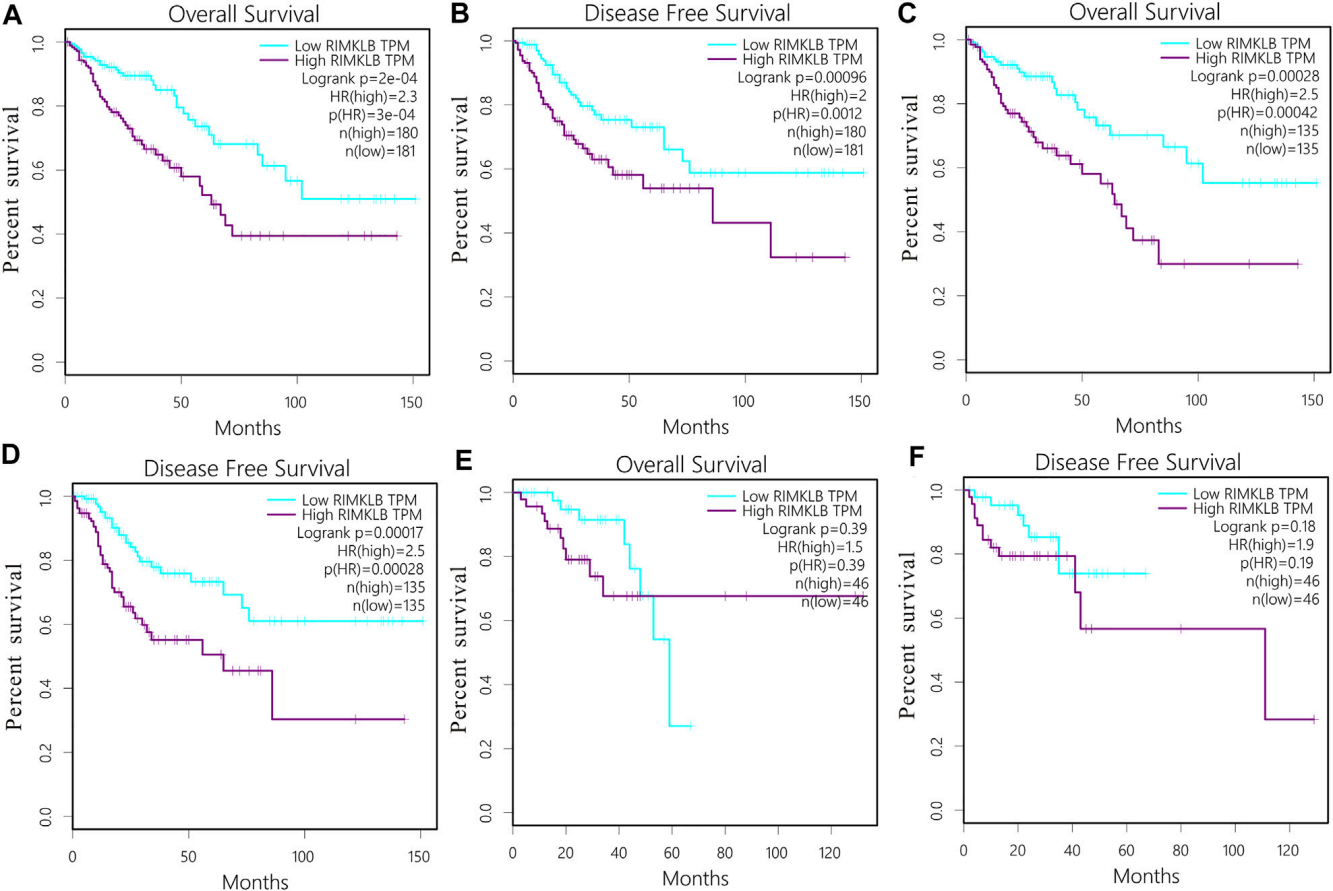
Survival curves and subgroup analysis of patients with colorectal cancer from TCGA cohort. Overall survival (OS) and disease free (DFS) curves comparing the high and low expression of RIMKLB in CRC **(A,B)**; OS and DFS curves comparing the high and low expression of RIMKLB in colon cancer **(C,D)** and rectum cancer **(E,F)**.

The prognostic significance of RIMKLB expression in CRC was analyzed using the TCGA RNA sequencing data from the GEPIA database. High RIMKLB expression levels were associated with poorer OS (HR = 2.3, *p* = 0.0003, Figure 3A), and DFS in CRC (HR = 2, *p* = 0.0012, Figure 3B). When subgrouped by tumor site, this association only existed in colon cancer (OS: HR = 2.5, P = 0.00042, Figure 3C; DFS: HR = 2.5, P = 0.00028, Figure 3D) and disappeared in rectal cancer (OS: HR = 1.5, P = 0.39, Figure 3E; DFS: HR = 1.9, P = 0.19, Figure 3F).

**FIGURE 4:**
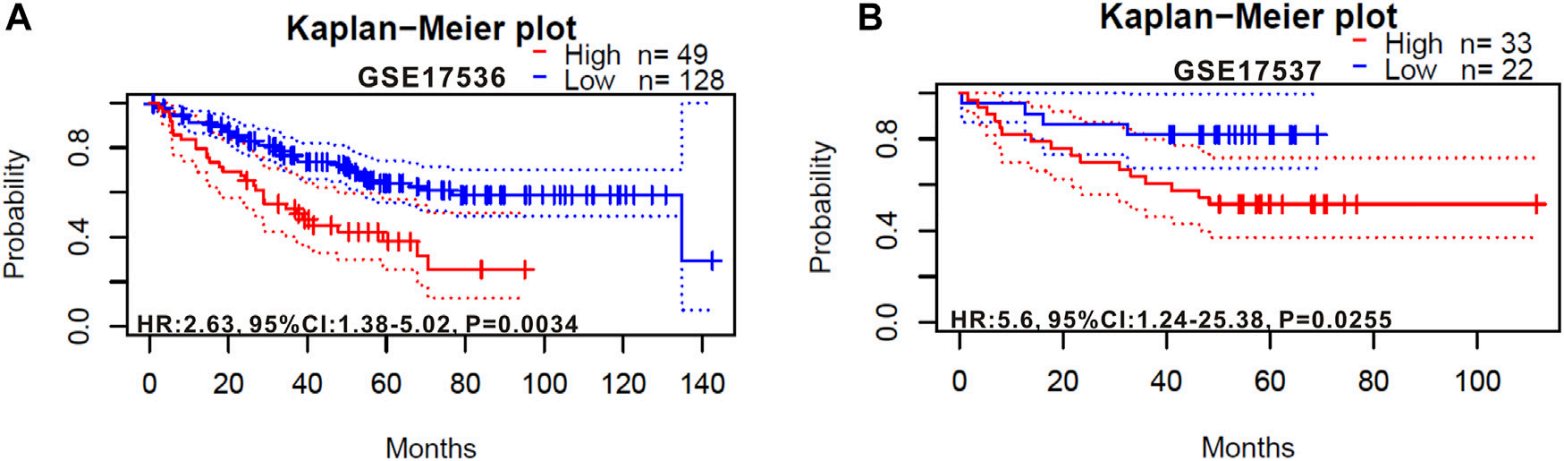
Kaplan-Meier survival curves comparing the high and low expression of RIMKLB in colorectal cancer in the PrognoScan. **(A)** Survival curves of OS colorectal cancer cohorts of GSE17536 (*n* = 177); **(B)** Survival curves of OS colorectal cancer cohorts of GSE17537 (*n* = 55).

We also verified the prognostic value of RIMKLB expression in CRC cancers using the Prognoscan website, whose data were from GEO database. High RIMKLB expression was associated with worse OS (HR = 2.63, 95% CI = 1.38–5.02, *p* = 0.0034, Figure 4A) among CRC patients in GSE17536, this survival significance (HR = 5.6, 95% CI = 1.24–25.36, *p* = 0.0255, Figure 4B) was also observed in GSE17537. In brief, high expression of RIMKLB is a potent risk factor among CRC patients.

### RimK-Like Family Member B Expression Levels Correlate With the Infiltration Levels of Immune Cells in Colorectal Cance

Previous studies have reported that survival time for colorectal cancers depends on the number and activity of tumor-infiltrating lymphocytes (Ohtani, 2007; Japanese Gastric Cancer Association, 2017). Therefore, we explored the relationship between RIMKLB expression with prognosis and the infiltrating immune cells in CRC using the TIMER and GEPIA database.

**FIGURE 5:**
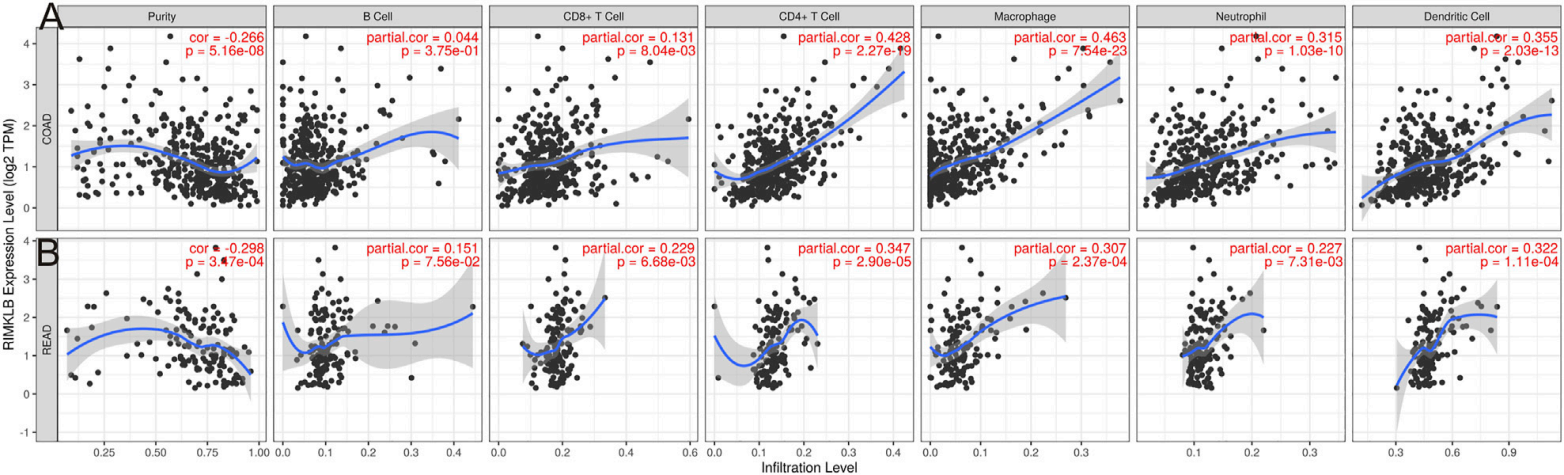
Correlation of RIMKLB expression with immune infiltration level in COAD (colon adenocarcinoma), and READ (recutm adenocarcinoma) in the TIMER database. **(A)** RIMKLB expression is significantly negatively related to tumor purity and has significant positive correlations with infiltrating levels of CD8^+^ T cells, CD4^+^ T cells, macrophages, neutrophils, and dendritic cells in COAD, other than B cells. **(B)** RIMKLB expression is significantly negatively related to tumor purity and has significant positive correlations with infiltrating levels of B cells, CD8^+^ T cells, CD4^+^ T cells, macrophages, neutrophils, and dendritic cells in READ.

The level of IMKLB expression positively correlated with the infiltration levels of CD8^+^ T cells (*r* = 0.131, *p* = 8.04e-03), CD4^+^ T (*r* = 0.428, *p* = 2.27e-19) cells, macrophages (*r* = 0.463, *p* = 7.54e-23), neutrophils (*r* = 0.315, *p* = 1.03e-10), and dendritic cells (*r* = 0.355, *p* = 2.03e-13), but negatively related to tumor purity (*r* = −0.266, *p* = 5.16e-08) and B cells (*r* = −0.044, *p* = 3.57e-01) in Colon adenocarcinoma (COAD) tissues (Figure 5A); The level of RIMKLB expression is significantly negatively related to tumor purity (*r* = −0.298, *p* = 3.47e-04) and has significant positive correlations with infiltrating levels of B cells (r = 0.151, *p* = 7.56e-02), CD8^+^ T cells (*r* = 0.229, *p* = 6.68e-03), CD4^+^ T cells (*r* = 0.347, *p* = 2.90e-05), macrophages (r = 0.307, *p* = 2.37e-04), neutrophils (r = 0.227, *p* = 7.31e-03), and dendritic cells (r = 0.322, *p* = 1.11e-04) in Rectum adenocarcinoma (READ) tissues (Figure 5B).

### Correlation of RimK-Like Family Member B Expression With Immune Checkpoint in COAD and READ.

**FIGURE 6:**
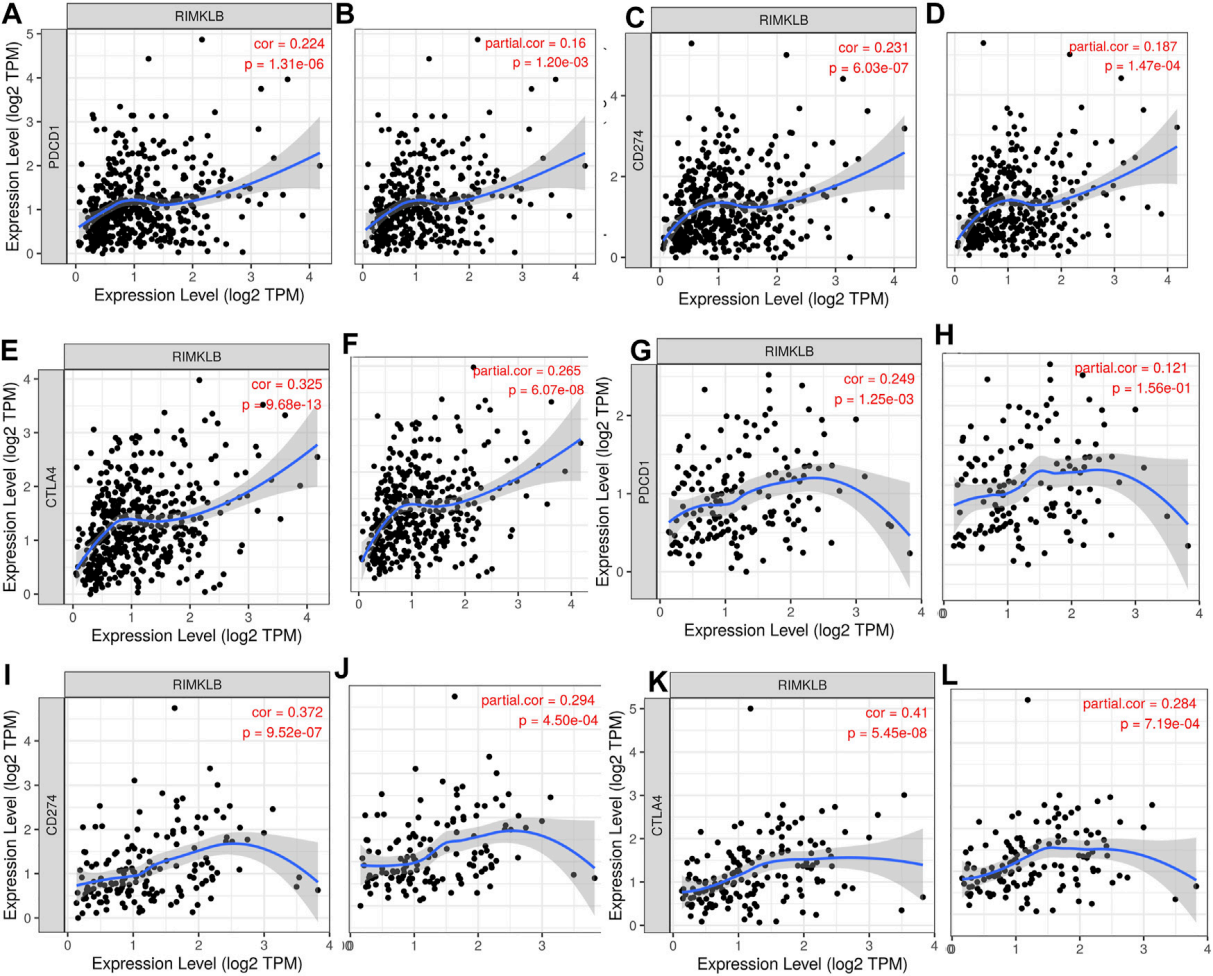
Correlation of RIMKLB expression with PD1, PDL1, and CTLA4 in COAD, and READ. RIMKLB expression has significant positive correlations with infiltrating levels of PD1 **(A,B)**, PDL1 **(C,D)** and CTLA4 **(E,F)** before and after purity adjustment in COAD; As well as in READ [PD1 **(G,H)**, PDL1 **(I,J)** and CTLA4 **(K,L)**].

Research on the role of targeted immune therapy in the treatment of advanced colorectal cancer and its relationship with tumor gene mutations has received more and more attention (Galon et al., 2007). Therefore, we used TIMER database to investigate the relationship between RIMKLB expression and immunotherapeutic targets in colorectal cancer. The results showed that the expression of RIMKLB was significantly correlated with it. We find that RIMKLB expression has significant positive correlations with infiltrating levels of PD1 (A, *r* = 0.223, *p* = 1.31e-06; B, *r* = 0.16, *p* = 1.20e-03), PDL1 (C, *r* = 0.223, *p* = 6.03e-07; D, *r* = 0.187, *p* = 1.47e-04) and CTLA4 (E, *r* = 0.325, *p* = 9.68e-13; F, *r* = 0.265, *p* = 6.07e-08) before and after purity adjustment in COAD; In addition, RIMKLB expression also has significant positive correlations with infiltrating levels of PD1 (G, *r* = 0.249, *p* = 1.25e-03; H, *r* = 0.121, *p* = 0.156e-01), PDL1 (I, *r* = 0.372, *p* = 9.52e-07; J, *r* = 2.94, *p* = 4.50e-04) and CTLA4 (K, *r* = 0.41, *p* = 5.45e-08; L, *r* = 0.284, *p* = 7.19e-04) before and after purity adjustment in READ (Figure 6A–L).

### Correlation Between RimK-Like Family Member B mRNA Levels and Different Subsets of Immune Cells

**FIGURE 7:**
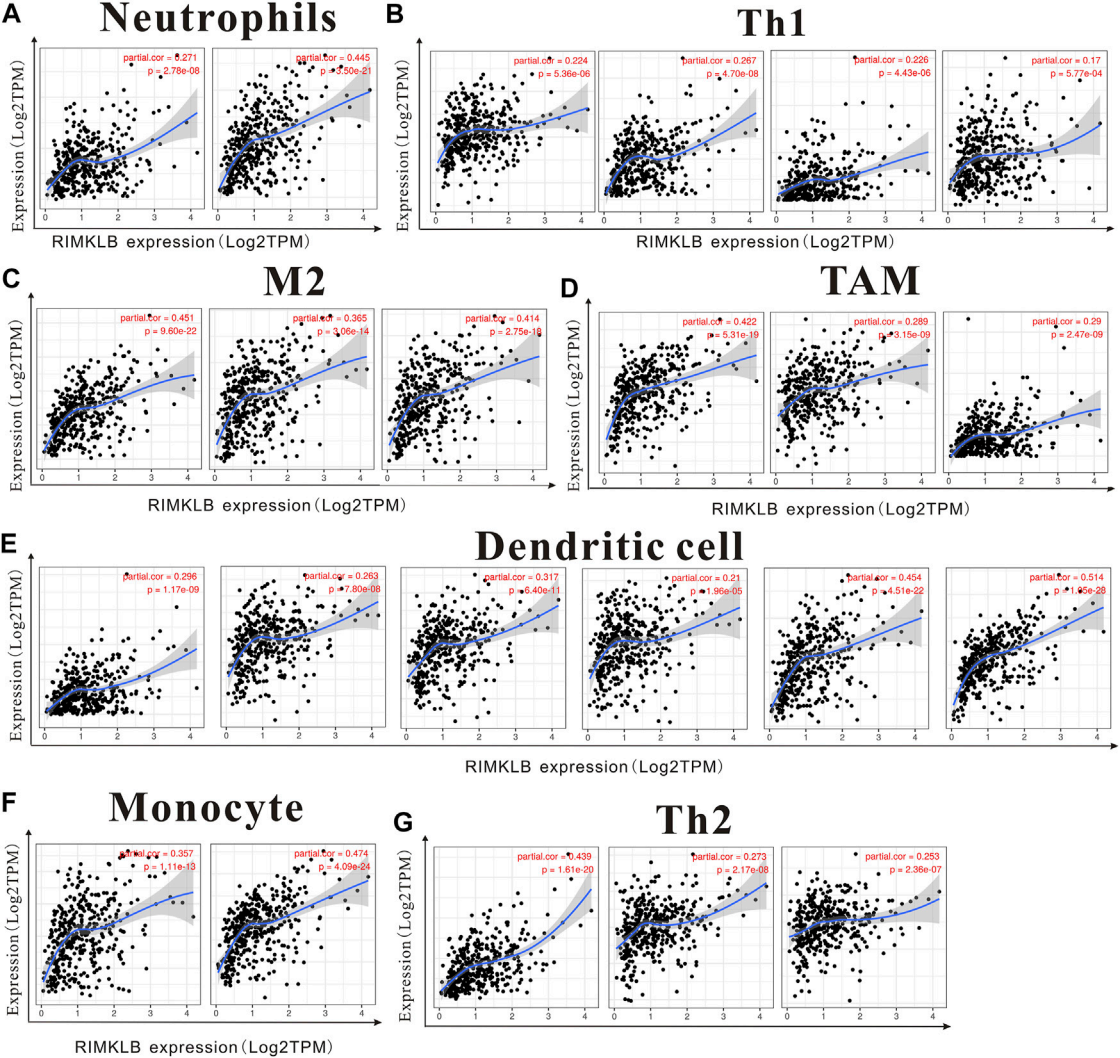
The expression of RIMKLB and the correlation with immune infiltration in COAD (colon adenocarcinoma). Markers include ITGAM, and CCR7 of Neutrophils; TBX21, STAT1, STAT4, and TNF of Th1; CD86 and CSF1R of monocytes; CD163, and VSIG4 of M2 macrophages; CCL2, CD68, and IL10 of TAMs (tumor-associated macrophages); HLA-DPB1, HLA-DRA, HLA-DPA1, CD1C, NRP, and ITGAX of Dendritic cell; GATA3, GATA6, and GATA5A of Th2. **(A–G)** Scatterplots of correlations between RIMKLB expression and gene markers of Neutrophils **(A)**, Th1 **(B)**, and M2 macrophages **(C)**, TAMs **(D)**, Dendritic cell **(E)**, monocytes **(F)**, and Th2 in COAD **(G)**.

**FIGURE 8:**
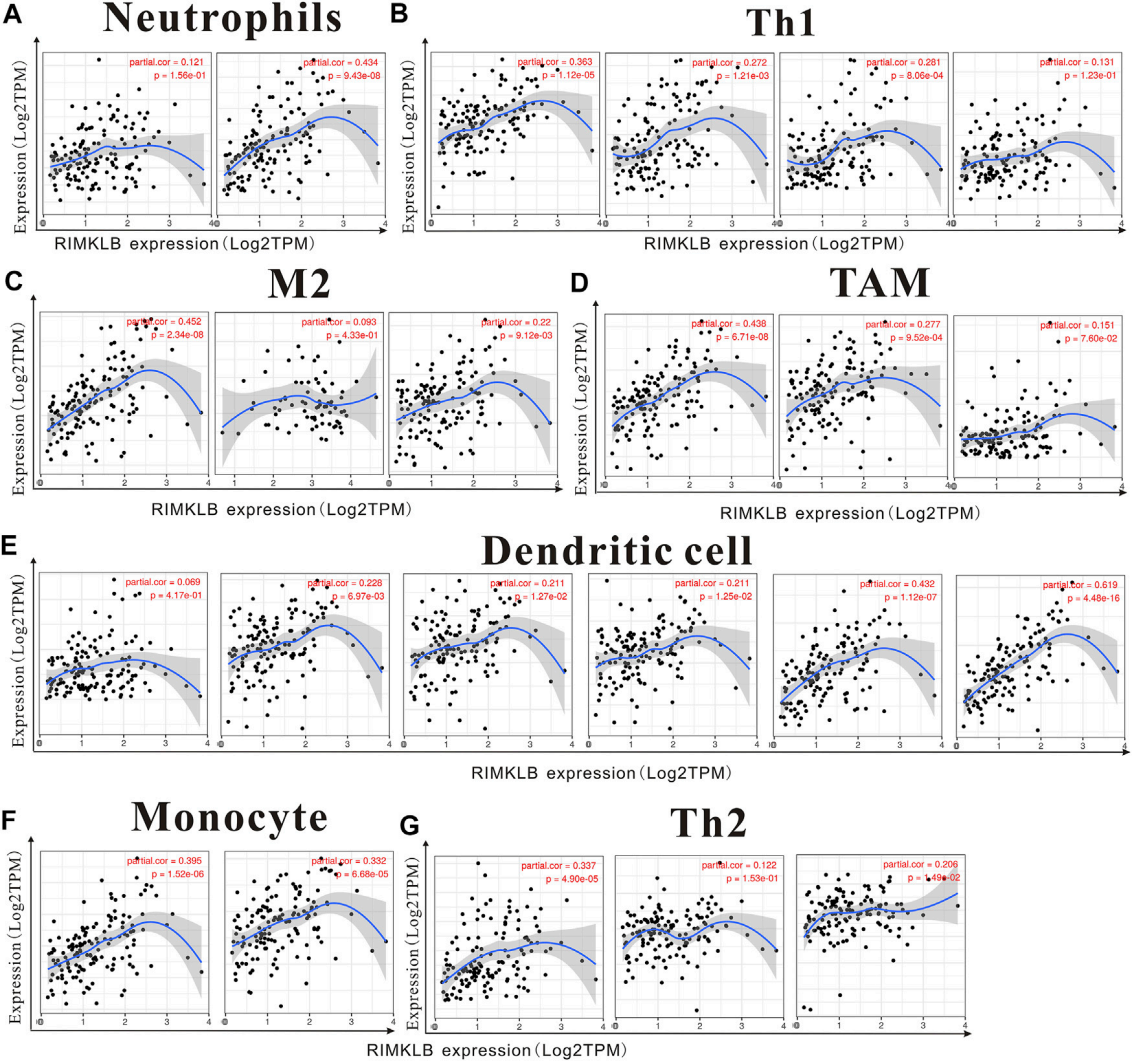
The expression of RIMKLB and the correlation with immune infiltration in READ (recutm adenocarcinoma). Markers include ITGAM, and CCR7 of Neutrophils; TBX21, STAT1, STAT4, and TNF of Th1; CD86 and CSF1R of monocytes; CD163, and VSIG4 of M2 macrophages; CCL2, CD68, and IL10 of TAMs (tumor-associated macrophages); HLA-DPB1, HLA-DRA, HLA-DPA1, CD1C, NRP, and ITGAX of Dendritic cell; GATA3, GATA6, and GATA5A of Th2. **(A–G)** Scatterplots of correlations between RIMKLB expression and gene markers of Neutrophils **(A)**, Th1 **(B)**, and M2 macrophages **(C)**, TAMs **(D)**, Dendritic cell **(E)**, monocytes **(F)**, and Th2 in COAD.

We used TIMER and GEPIA databases to investigate the relationship between RIMKLB and various immune-infiltrating cells based on the expression levels of immune marker genes in colon and rectum tissues. The immune cells analyzed in CRC tissues included CD8^+^ T cells, CD4^+^ T cells, B cells, tumor-associated macrophages (TAMs), monocytes, M1 and M2 macrophages, neutrophils, and natural killer (NK) cells, dendritic cells (DCs), subsets of T cells [T helper 1 (Th1), Th2, follicular helper T (Tfh), Th17]. The results showed that RIMKLB expression level was significantly correlated with most immune marker groups of various immune cells and different T cells in colon and rectum cancer (Table 1). Interestingly, we found that the expression levels of marker sets of Neutrophils, Th1, M2 macrophages, TAMs, Dendritic cell, monocytes, and Th2 have strong correlations with RIMKLB expression in colon and rectum. The correlation analysis was adjusted for purity because tumor purity of clinical samples affected the analysis of immune infiltration (Table 2). To be specific, we showed that ITGAM, and CCR7 of Neutrophils; TBX21, STAT1, STAT4, and TNF of Th1; CD86 and CSF1R of monocytes; CD163, and VSIG4 of M2 macrophages; CCL2, CD68, and IL10 of TAMs (tumor-associated macrophages); HLA-DPB1, HLA-DRA, HLA-DPA1, CD1C, NRP, and ITGAX of Dendritic cell; GATA3, GATA6, and GATA5A of Th2 were significant correlated with RIMKLB expression in COAD (*p* < 0.0001; Figures 7A–G) and READ (*p* < 0.0001; Figures 8A–G).

**TABLE 1:** Correlation analysis between RIMKLB and related gene markers of immune cells in COAD and READ.

Cell Type	Marker	COAD				READ			
		None		Purity		None		Purity	
		Cor*	*p* Value	Cor	*p* Value	Cor*	*p* Value	Cor	*p* Value
CD8^+^ T cell	CD8A	0.203	1.22E-05	0.129	9.43E-03	0.342	7.64E-06	0.237	5.00E-03
	CD8B	0.178	1.34E-04	0.157	1.51E-03	0.204	8.49E-03	0.112	1.90E-01
T cell (general)	CD3D	0.202	1.34E-05	0.131	8.16E-03	0.191	1.39E-02	0.048	5.71E-01
	CD3E	0.28	1.29E-09	0.218	9.24E-06	0.285	2.10E-04	0.159	6.18E-02
	CD2	0.259	2.12E-08	0.185	1.73E-04	0.321	2.85E-05	0.206	1.51E-02
B cell	CD19	0.233	4.42E-07	0.15	2.43E-03	0.087	2.632–01	0.01	9.11E-01
	CD79A	0.278	1.62E-09	0.186	1.59E-04	0.194	1.22E-02	0.054	5.28E-01
Monocyte	CD86	0.404	0.00E + 00	0.357	1.11E-13	0.478	9.65E-11	0.395	1.52E-06
	CD115	0.491	0.00E + 00	0.474	4.09E-24	0.423	1.95E-08	0.332	6.68E-05
TAM	CCL2	0.463	0.00E + 00	0.422	5.31E-19	0.515	0.00E + 00	0.438	6.71E-08
	CD68	0.32	2.16E-12	0.289	3.15E-09	0.357	2.80E-06	0.277	9.52E-04
	IL10	0.324	1.20E-12	0.29	2.47E-09	0.233	2.57E-03	0.151	7.60E-02
M1 macrophage	INOS	−0.194	3.09E-05	−0.23	2.26E-06	−0.085	2.78E-01	−0.044	6.05E-01
	IRF5	0.336	1.92E-13	0.354	1.91E-13	0.237	2.16E-03	0.203	1.66E-02
	COX2	0.187	5.59E-05	0.12	1.54E-02	0.256	9.20E-04	0.159	6.11E-02
M2 macrophage	CD163	0.474	0.00E + 00	0.451	9.60E-22	0.529	0.00E + 00	0.452	2.34E-08
	VSIG4	0.443	0.00E + 00	0.414	2.75E-18	0.314	4.20E-05	0.22	9.12E-03
	MS4A4A	0.403	0.00E + 00	0.365	3.06E-14	0.455	1.11E + 09	0.093	4.33E-01
Neutrophils	CD66b	−0.111	1.73E-02	−0.12	1.67E-02	−0.379	4.84E-07	−0.3	3.47E-04
	CD11b	0.468	0.00E + 00	0.445	3.50E-21	0.526	0.00E + 00	0.434	9.43E-08
	CCR7	0.338	1.34E-13	0.271	2.78E-08	0.187	1.61E-02	0.121	1.56E-01
Natural killer cell	KIR2DL1	0.08	8.72E-02	0.034	4.95E-01	0.186	1.66E-02	0.166	5.02E-02
	KIR2DL3	0.085	6.85E-02	0.075	1.31E-01	0.182	1.88E-02	0.174	4.07E-02
	KIR2DL4	−0.023	6.23E-01	−0.1	4.33E-02	0.1	2.01E-01	−0.032	7.12E-01
	KIR3DL1	0.104	2.60E-02	0.051	3.04E-01	0.08	3.08E-01	0.041	6.34E-01
	KIR3DL2	0.136	3.63E-03	0.076	1.28E-01	0.199	1.01E-02	0.114	1.83E-01
	KIR3DL3	−0.061	1.89E-01	−0.06	2.38E-01	−0.051	5.11E-01	−0.099	2.48E-01
	KIR2DS4	0.033	4.86E-01	0.016	7.47E-01	0.065	4.07E-01	−0.027	7.48E-01
Dendritic cell	HLA-DPB1	0.37	2.33E-16	0.317	6.40E-11	0.331	1.52E-05	0.211	1.27E-02
	HLA-DQB1	0.218	2.61E-06	0.158	1.40E-03	0.097	2.12E-01	0.041	6.35E-01
	HLA-DRA	0.271	4.33E-09	0.21	1.96E-05	0.325	2.11E-05	0.211	1.25E-02
	HLA-DPA1	0.32	2.29E-12	0.263	7.80E-08	0.355	3.07–06	0.228	6.97E-03
	BDCA-1	0.339	9.49E-14	0.296	1.17E-09	0.195	1.16E-02	0.069	4.17E-01
	BDCA-4	0.546	0.00E + 00	0.514	1.05E-28	0.672	0.00E + 00	0.619	4.48E-16
	CD11c	0.484	0.00E + 00	0.454	4.51E-22	0.496	5.97E-12	0.432	1.12E-07
Th1	T-bet	0.267	6.68E-09	0.226	4.43E-06	0.367	1.18E-06	0.281	8.06E-04
	STAT4	0.308	2.00E-11	0.267	4.70E-08	0.332	1.37E-05	0.272	1.21E-03
	STAT1	0.258	2.02E-08	0.224	5.36E-06	0.445	2.84E-09	0.363	1.12E-05
	IFN-γ	0.077	9.99E-02	0.041	4.11E-01	0.301	7.96E-05	0.209	1.34E-02
	TNF-α	0.21	5.90E-06	0.17	5.77E-04	0.226	3.41E-03	0.131	1.23E-01
Th2	GATA3	0.486	1.52E-28	0.439	1.61E-20	0.422	1.99E-08	0.337	4.90E-05
	STAT6	0.247	8.08E-08	0.253	2.36E-07	0.181	2.01E-02	0.206	1.49E-02
	STAT5A	0.271	4.36E-09	0.273	2.17E-08	0.137	7.94E-02	0.122	1.53E-01
	IL13	0.161	5.62E-04	0.104	3.59E-02	0.127	1.02E-01	−0.008	9.22E-01
Tfh	BCL6	0.459	0.00E + 00	0.425	3.27E-19	0.597	0.00E + 00	0.595	1.18E-14
	IL21	0.102	2.85E-02	0.066	1.81E-01	0.061	4.43E-01	0.036	6.73E-01
Th17	STAT3	0.246	1.02E-07	−0.27	5.16E-08	0.403	9.86E-08	0.339	3.47E-04
	IL17A	−0.157	7.46E-04	−0.16	1.35E-03	−0.21	6.60E-03	−0.186	2.82E-02

Cor*: Correlation.

**TABLE 2:** Correlation analysis between RIMKLB and significant gene markers of immune cells in GEPIA.

Description	Gene Markers	COAD		READ	
		Correlation	*p* Value	Correlation	*p* Value
Monocyte	CD86	0.42	2.20E-13	0.27	0.01
	CD115 (CSF1R)	0.51	0	0.29	0.0046
TAM	CCL2	0.53	0	0.25	0.015
	CD68	0.27	5.30E-06	0.19	0.076
	IL10	0.38	7.40E-11	0.2	0.056
M2 Macrophage	CD163	0.43	1.70E-13	0.3	0.0036
	VSIG4	0.44	3.20E-14	0.23	0.03
	MS4A4A	0.47	0	0.26	0.013
Neutrophils	CD11b (ITGAM)	0.47	4.40E-16	0.25	0.018
	CCR7	0.47	0	0.078	0.46
Dendritic cell	HLA-DPB1	0.31	2.00E-07	0.24	0.023
	HLA-DRA	0.25	1.90E-05	0.19	0.075
	HLA-DPA1	0.29	1.10E-06	0.2	0.057
	BDCA-1(CD1C)	0.41	1.60E-12	0.055	0.6
	BDCA-4(NRP1)	0.58	0	0.3	0.0041
	CD11c (ITGAX)	0.42	6.70E-13	0.21	0.044
Th1	T-bet (TBX21)	0.31	1.40E-07	0.18	0.089
	STAT4	0.45	7.30E-15	0.2	0.06
	STAT1	0.22	0.00027	0.19	0.072
	TNF-α (TNF)	0.23	0.00013	0.14	0.18
Th2	GATA3	0.58	0	0.18	0.085
	STAT6	0.15	0.01	0.26	0.013
	STAT5A	0.35	1.70E-09	0.085	0.42

### Co-Expression and Correlation Amongst the Other Genes Associated With RimK-Like Family Member B in Colorectal Cancer

The top 25 positively co-expressed genes were analyzed *via* cBioPortal, containing the Spearman’s correlation coefficient, *p*-value from two-sided *t*-test, and also *q*-value derived from the Benjamini–Hochberg FDR correction procedure (Table 3). The correlation graph was obtained using the Pearson’s correlation coefficient amongst RIMKLB gene with AKT3 (*r*-value- 0.68), MPDZ (*r*-value-0.66), PKD2 (*r*-value- 0.67) and MAP1B (R-value- 0.69) (Supplementary Figures S1A–S1D). Collectively all these results reveal that the RIMKLB gene has a positive association and correlation with AKT3, MPDZ, PKD2, and MAP1B to upregulate the gene expression to induce the development of colorectal cancer.

**TABLE 3:** TOP genes positively correlated with RIMKLB in CRC.

Correlated gene	Cytoband	Spearman’s correlation	*p*-Value	q-Value
AKT3	1q43-q44	0.706	2.12E-80	4.21E-76
MPDZ	9p23	0.689	4.35E-75	4.32E-71
PKD2	4q22.1	0.681	1.05E-72	6.96E-69
MAP1B	5q13.2	0.679	5.94E-72	2.95E-68
LHFPL6	13q13.3-q14.11	0.674	1.09E-70	4.35E-67
MEIS1	2p14	0.673	1.86E-70	5.52E-67
DNAAF9	20p13	0.673	1.95E-70	5.52E-67
BNC2	9p22.3-p22.2	0.669	2.57E-69	6.39E-66
TNS1	2q35	0.665	4.52E-68	9.97E-65
SLIT2	4p15.31	0.664	5.52E-68	1.10E-64
FBXL7	5p15.1	0.664	6.19E-68	1.12E-64
AMOTL1	11q21	0.663	1.28E-67	2.12E-64
DZIP1	13q32.1	0.662	2.38E-67	3.64E-64
HEG1	3q21.2	0.661	3.57E-67	5.07E-64
ARHGEF25	12q13.3	0.661	5.62E-67	7.44E-64
PTPRM	18p11.23	0.659	1.81E-66	2.25E-63
ZEB1	10p11.22	0.655	1.47E-65	1.72E-62
FILIP1	6q14.1	0.653	6.50E-65	7.17E-62
MCC	5q22.2	0.652	1.24E-64	1.30E-61
JAM3	11q25	0.651	1.69E-64	1.68E-61
TUB	11p15.4	0.648	9.10E-64	8.61E-61
STON1	2p16.3	0.647	1.75E-63	1.58E-60
WHAMMP2	15q13.1	0.646	2.66E-63	2.30E-60
JCAD	10p11.23	0.646	2.85E-63	2.36E-60
SALL2	14q11.2	0.646	3.71E-63	2.95E-60

### The Mutational Analysis of RimK-Like Family Member B in Colorectal Cancer

The RIMKLB gene mutation was analyzed on COSMIC database comprising more than 2,406 samples of colorectal cancer out of which 77 were recorded for mutations, among them the missense substitution is highest with 53.25% followed by synonymous substitution (23.38%), nonsense substitution (1.30%) and other types (6.49%) (Supplementary Figure S2A).

The breakdown of various substitution mutation is shown in Supplementary Figure S2B, representing the highest type of G > A (39.66%) and lowest showing T > A (3.45%).

To determine and analyze the frequency and type of mutation, cBioPortal server was used where the cancer type summary indicates the mutation along with the various subtypes of colorectal cancer showing mucinous adenocarcinoma of colon and rectum (>6%), colon adenocarcinoma (<6%), and rectal adenocarcinoma (∼2%) (Supplementary Figure S2C). The Oncoprint and Mutation tab shows that the RIMKLB gene is altered in 2.5% of the total patients in TCGA colorectal cancer dataset along with the heatmap for the associated genes (Supplementary Figure S2D). Additionally, a mutational study for the correlation among the RIMKLB gene with AKT3, MPDZ, PKD2, and MAP1B (Supplementary Figures S3A–S3D) showing a significant coefficient value for both Spearman and Pearson Correlation test and the regression line. It is observed that the mutation of RIMKLB is much more expressive for AKT3 > MPDZ > PKD2 > MAP1B.

### RimK-Like Family Member B-Related Biological Pathways in Patients With Colorectal Cancer

We carried out the biological process and KEGG pathway to further investigate the potential pathways of RIMKLB in CRC. RIMKLB was mainly involved in cell-cell adhesion *via* plasma-membrane adhesion molecules, humoral immune response mediated by circulating immunoglobulin, cytolysis, killing by host of symbiont cells, triglyceride-rich lipoprotein particle remodeling, regulation of intestinal absorption, chylomicron assembly (Supplementary Figure S6A). Moreover, KEGG analysis revealed that RIMKLB is involved in pathways of ECM-receptor interaction, Cell adhesion molecules, Platelet activation, Chemical carcinogenesis-DNA adducts, cAMP signaling pathway, PI3K-Akt signaling pathway, and Cytokine-cytokine receptor interaction. RIMKLB is associated with local immunity in colorectal cancer, and its abnormal expression may lead to the occurrence and development of CRC (Supplementary Figure S5B).

## Disscussion

**FIGURE 9:**
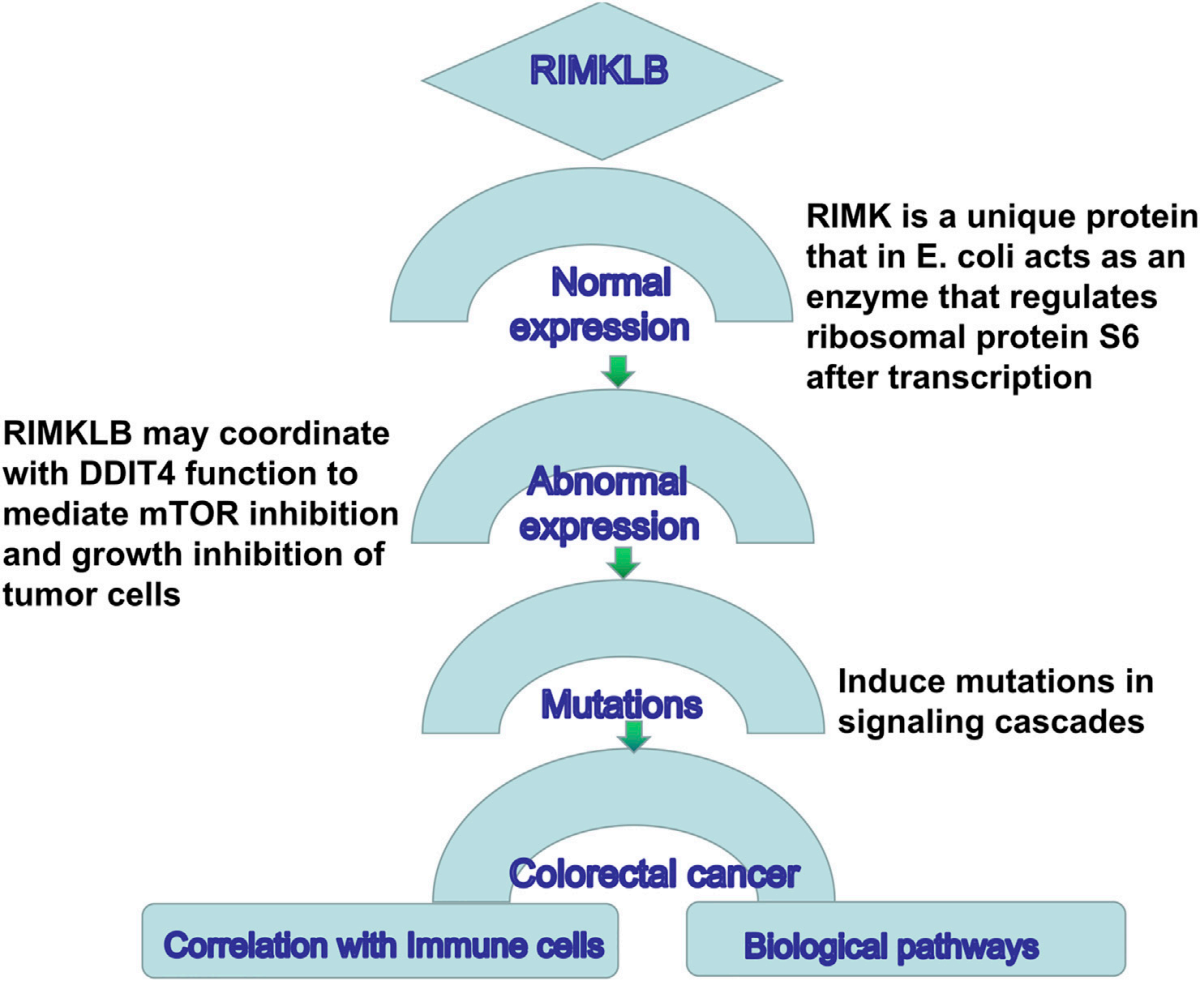
Schematic representation for functional relevance of RIMKLB gene in the oncogenesis of colorectal cancer and its candidature as a correlation with immune cells and biological pathways.

This study was the first to reveal the expression and prognostic efficacy of RIMKLB in CRC. We found that the expression of this gene was significantly different in a variety of tumors. Notably, it was significantly decreased in CRC tumors compared to normal tissues, and this was correlated with histology, stage, lymph node metastasis, and tumor status. Moreover, multiple databases confirmed that a high expression of RIMKLB was associated with worse OS and DFS, indicating that this gene may play an important role in tumor development. The Schematic representation for functional relevance of RIMKLB gene in the oncogenesis of colorectal cancer and its candidature as a correlation with immune cells and biological pathways is in Figure 9.

As far as we know, there are very few studies on RIMKLB and Immune infiltration at present. This is the first study to find a close correlation between RIMKLB and immune infiltration in CRC. TIMER analysis showed that the mRNA level of RIMKLB was closely related to TIICs, including CD4^+^ and CD8^+^ T cells, B cells, TAMs M1 and M2 macrophages, neutrophils, monocytes, natural killer cells, dendritic cells, Th1, Th2, Tfh, and Th17. In addition, the expression of RIMKLB expression was significantly correlated with the infiltration level of immune checkpoint inhibitors (ICIs), and enrichment analysis showed that RIMKLB was positively correlated with immunoinflammatory pathways. This study explored the correlation between RIMKLB and the immune microenvironment, providing new ideas and targets for CRC immunotherapy.

Our study found that there were differences in the expression of RIMKLB between COAD and READ. High expression of RIMKLB in rectal cancer indicated poor OS and DFS, while there was no significant statistical correlation in the case of colon cancer. In COAD and READ, the expression of RIMKLB maintained a high degree of consistency with tumor immune cell infiltration and the expression of immune examination, except for B cell infiltration and PD1 expression. For this reason, we first examined the differences in sample size between COAD and READ groups, and secondly, the possible differences in the pathogenesis of the two cancer types. A retrospective analysis comparing right-sided colon cancer (RCC), left-sided colon cancer (LCC), and colorectal cancer with regards to tumor status, differentiation degree, infiltration depth and diameter showed that TNM staging and PFS of RCC was lower than that of the LCC and rectal cancer; hence, survival may be associated with inherent position characteristics (Gao et al., 2017). Studies have found that in addition to anatomical differences, RCC and LCC are also different in embryo origin and metastasis patterns and drug target composition (Tamas et al., 2015). Human colon and rectal cancers were comprehensively analyzed by the Cancer Genome Map Network to identify possible genetic differences between them. Research data show that non-high-mutation tumors correspond to CIN phenotype, while high-mutation tumors correspond to microsatellite instability (MSI) phenotype (Network, 2012). Studies have identified common tumor-initiating events involving APC, KRAS, and TP53 genes in RCC, LCC, and rectal cancer through comparative somatic and proteomic analyses of the three cancer types. However, The sequence of each event in tumor development and selection of downstream somatic changes is different at all three anatomic sites, which may have therapeutic relevance in these highly complex and heterogeneous tumors (Imperial et al., 2018). Therefore, in our study, the slight differences between the two may be closely related to the above reasons, which need further research and verification.

Immunotherapy is a new type of cancer treatment. The strategy is to use the patient’s own immune system to fight cancer cells. Tumor immunotherapy overcomes the major problem of specificity in chemotherapy and radiotherapy. Although immunotherapy has dramatically changed the treatment outlook for many advanced cancers, the benefits of CRC to date have been limited to patients with high microsatellite instability (MSI-H) DNA mismatched repair defect (dMMR) tumors, and several randomized controlled trials are under way to move immunotherapy to first-line and adjuvant therapy for metastatic cancers (Franke et al., 2019). In recent years, a lot of work has been done to evaluate the prognostic value of various immune cell subsets. In general, cytotoxic T cells, memory T cells, Th1 cells, Tfh cells and B cells are associated with prolonged survival, while increased density of Treg cells, myeloid-derived suppressor cells and neutrophils is associated with poor prognosis (Bruni et al., 2020). Similar results were found in our study. In our study, we found that in colon and rectal cancer, the expression level of RIMKLB was significantly correlated with most immune marker groups of various immune cells and different T cells. Interestingly, we found that neutrophils, Th1, M2 macrophages, TAM, DCs, monocytes, and Th2 were strongly correlated with the expression of RIMKLB expression in the colon and rectum. ICIs is used to target and/or block immune checkpoint protein ligands on the surface of T cells or other immune cell subsets in order to restore immune function. However, the high activation and overexpression of immune checkpoints in cancer lead to the suppression of anti-tumor immune response, which is conducive to the proliferation and diffusion of malignant cells (Pardoll, 2015; Gonzalez et al., 2018). ICIs, specifically PD-1, PDL-1 and CTLA-4 inhibitors, have been approved for the treatment of a variety of solid tumors. Pd-1 and CTLA-4 are both negative costimulatory molecules, and when inhibited, they enhance the activation of T cells and eventually kill tumor cells (Wei et al., 2018). ICIs can be used for tumors with MSI-H and high tumor mutational burden (TMB) in chemo-resistant environments. The most important biomarkers that should be routinely examined in clinical practice include PDL-1, MSI and TMB (Spencer et al., 2016; Mazloom et al., 2020). Our study found that the expression of RIMKLB was significantly correlated with ICIs, specifically with the infiltration levels of PD1, PD-L1, and CTLA4. At the same time, the enrichment analysis of GO pathway suggested that this gene was also involved in immune function. Taken together, these findings suggest that RIMKLB may be closely related to CRC immunotherapy, although further verification is needed.

The role of PI3K-Akt signaling pathway in the occurrence and progression of CRC and its important role in drug resistance have been reported earlier (Narayanankutty, 2019). Studies have confirmed that overexpression of IMPDH2 can promote cell G1/S phase cycle transition by activating the PI3K/AKT/mTOR and PI3K/AKT/FOXO1 pathways, and promote cell invasion, migration and EMT by regulating the PI3K/AKT/mTOR pathway (Narayanankutty, 2019). Han et al. (2020) found that loss of MLH1 reduced CTX sensitivity through HER-2/PI3K/AKT signal transduction and anti-apoptosis and induced activation of HER-2/PI3K/AKT signaling pathway, leading to cetuximab resistance in colon cancer. FAT4 can partially regulate PI3K activity to promote autophagy and inhibit EMT through PI3K/AKT/mTOR and PI3K/AKT/GSK-3β signaling pathways (Wei et al., 2019). Patra et al. (2021) found that COL11A1 plays an important role in regulating cell division, differentiation, proliferation, migration, growth and apoptosis of intestinal and colon cells, and it can disrupt a variety of signaling pathways that affect tumor development, such as RTK-RAS-PI3K, Wnt, TGF-β_2_ and TP53 pathways. At present, most studies have confirmed that the PI3K-Akt signaling pathway is regulated by multiple factors and plays a role in the occurrence, development and treatment of tumors. In our study, we found that RIMKLB is enriched in the PI3K-Akt pathway, suggesting that this molecule plays a role in CRC progression or treatment, but the specific mechanism needs further experimental verification.

Our research has its limitations. First of all, our study lacks cytological and animal experiments, and the specific mechanism is not clear. Further molecular cytological studies are needed in the future. Second, our retrospective study and small sample size failed to obtain immunotherapy data for these patients; Finally, there is a lack of data on molecular indicators (such as MSI, TP53, and TMB, etc) associated with colorectal cancer prognosis and immunotherapy, so further improvement is needed.

## Conclusion

Our study revealed the relationship between RIMKLB and the prognosis of CRC for the first time, and also found that this molecule was closely related to the invasion of CRC immune cells and ICIs. Thus, our study provides an important basis for the immunotherapy of CRC, the mechanism of immune resistance, and the identification of new immune-related therapeutic targets.

## Data Availability

The original contributions presented in the study are included in the article/Supplementary Material, further inquiries can be directed to the corresponding authors.
