# Supplementary figures and images for: The Prognostic Significance of RIMKLB and Related Immune Infiltrates in Colorectal Cancers
Source: Front Genet. 2022 Apr 4;13:818994. doi: 10.3389/fgene.2022.818994 (PMC9015428; doi:10.3389/fgene.2022.818994)

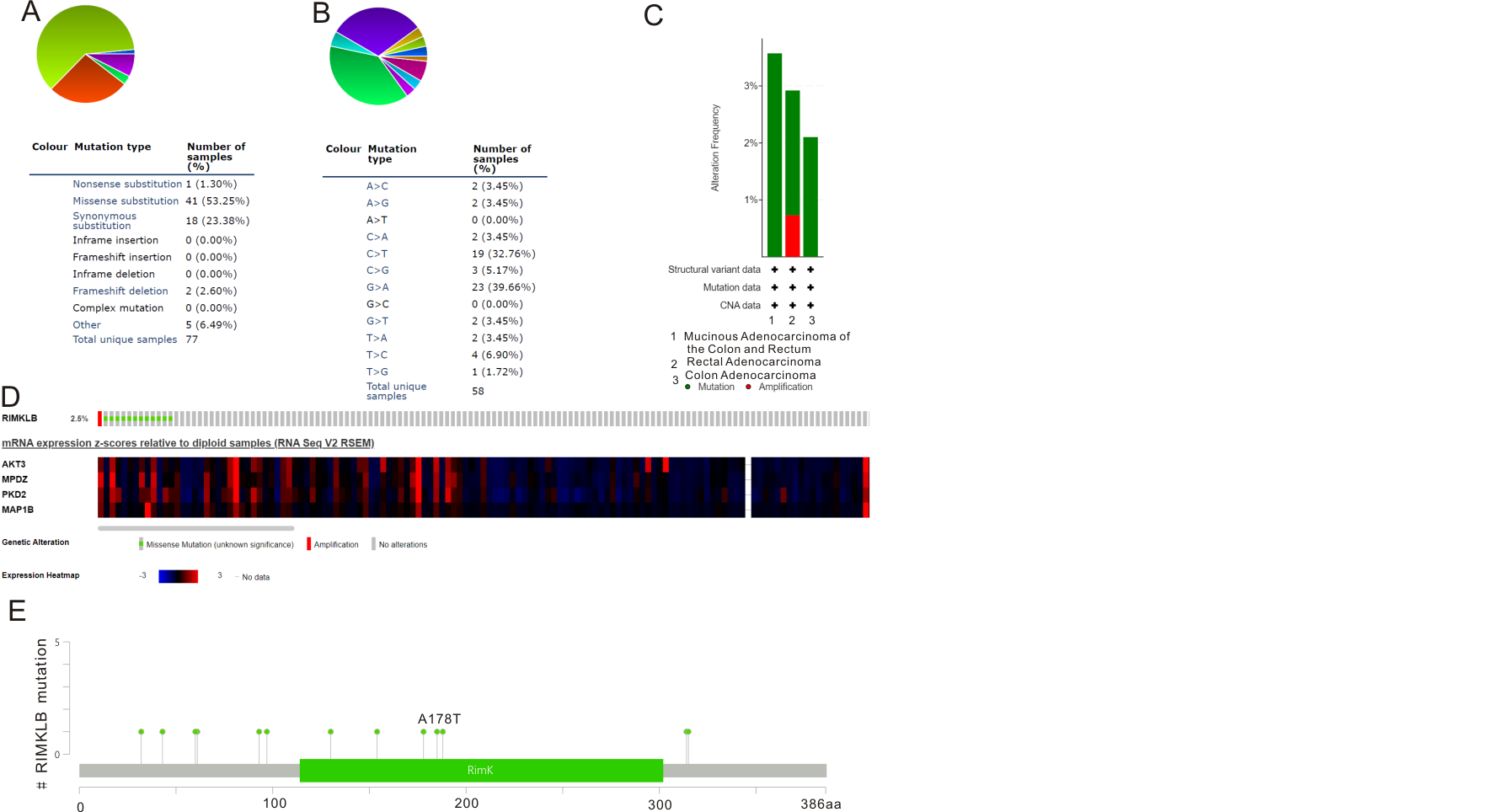

Supplement: Supplementary file 1 [file Image3.TIF]

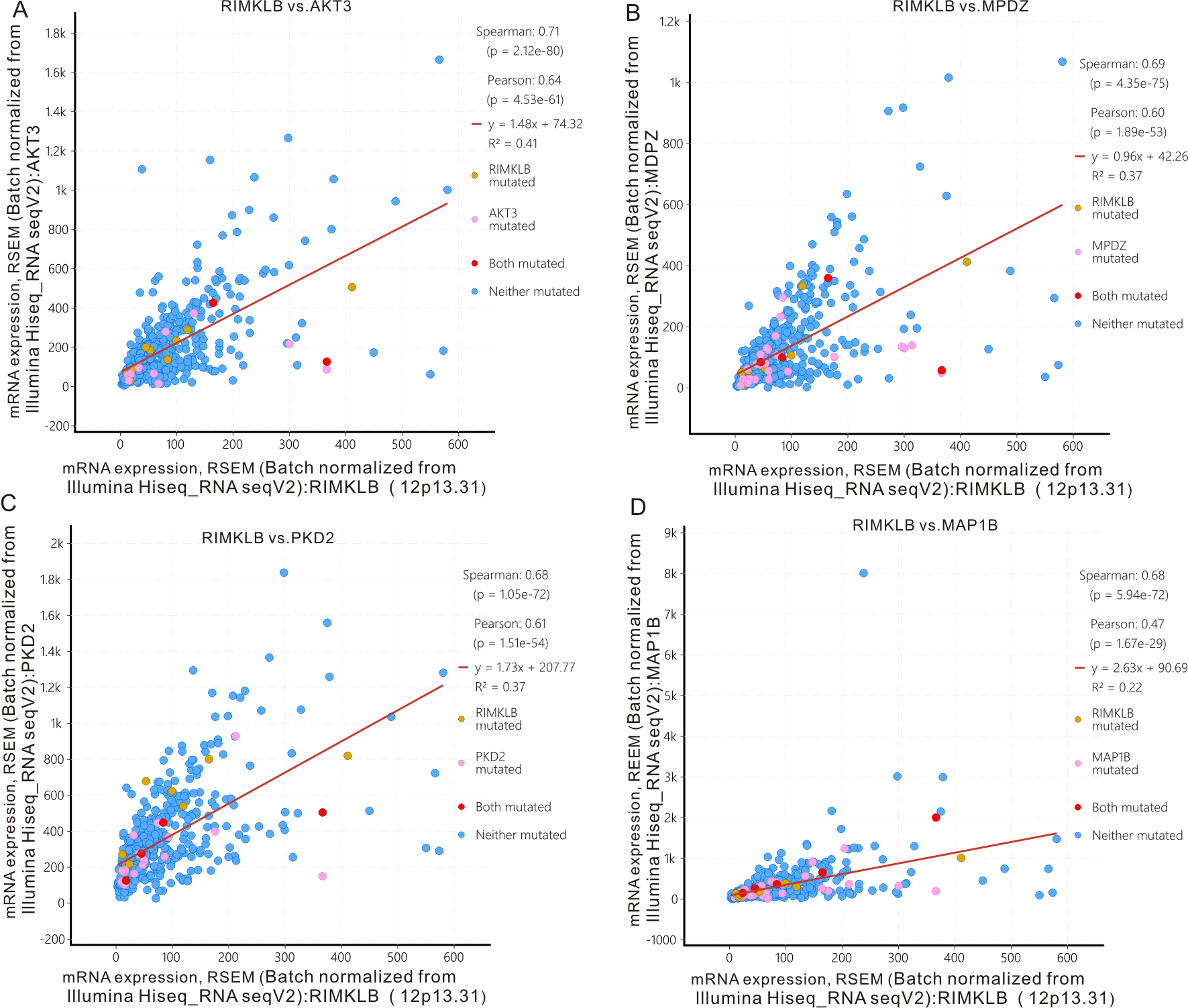

Supplement: Supplementary file 2 [file Image4.TIF]

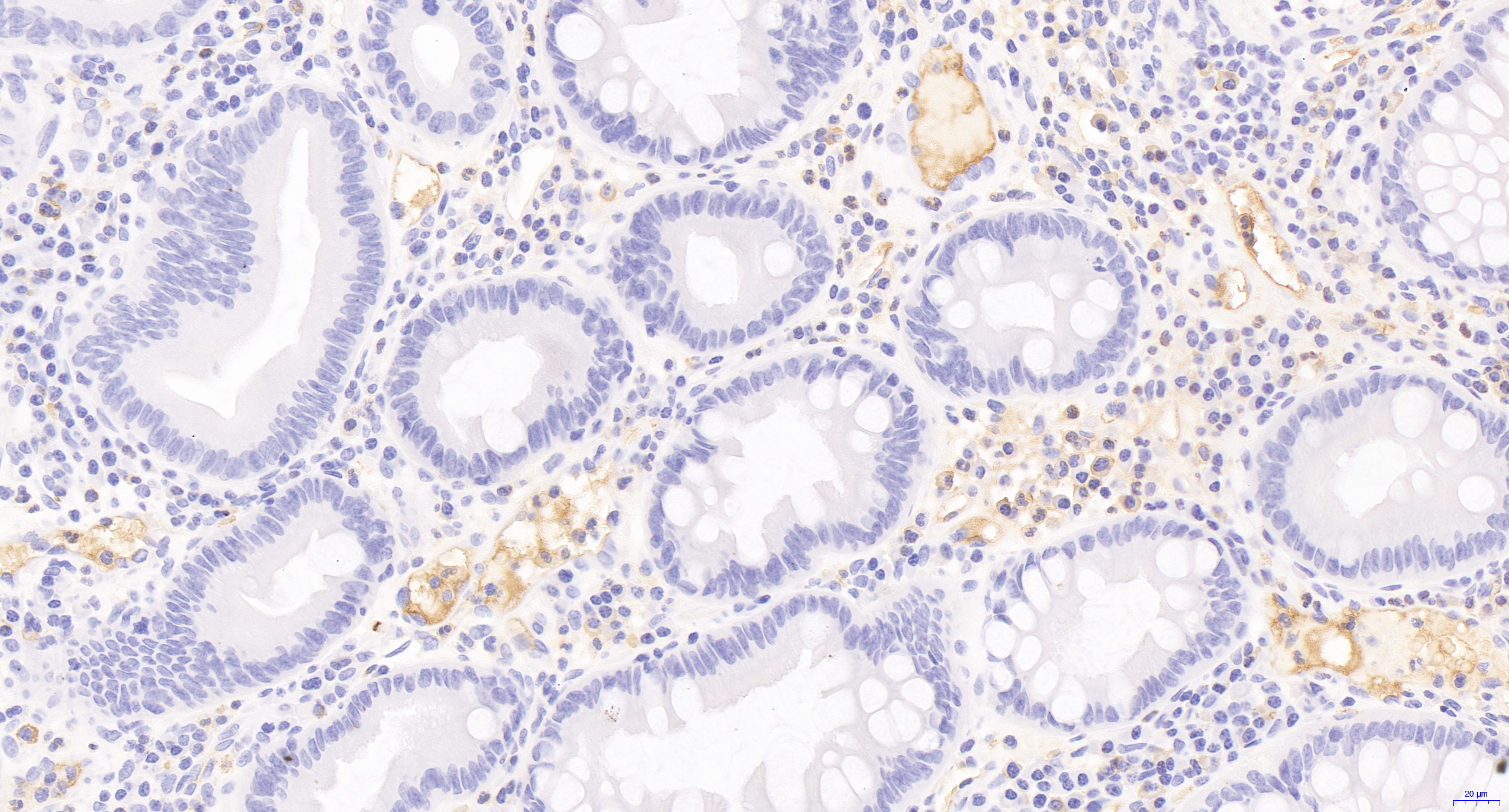

Supplement: Supplementary file 3 [file Image1.JPEG]

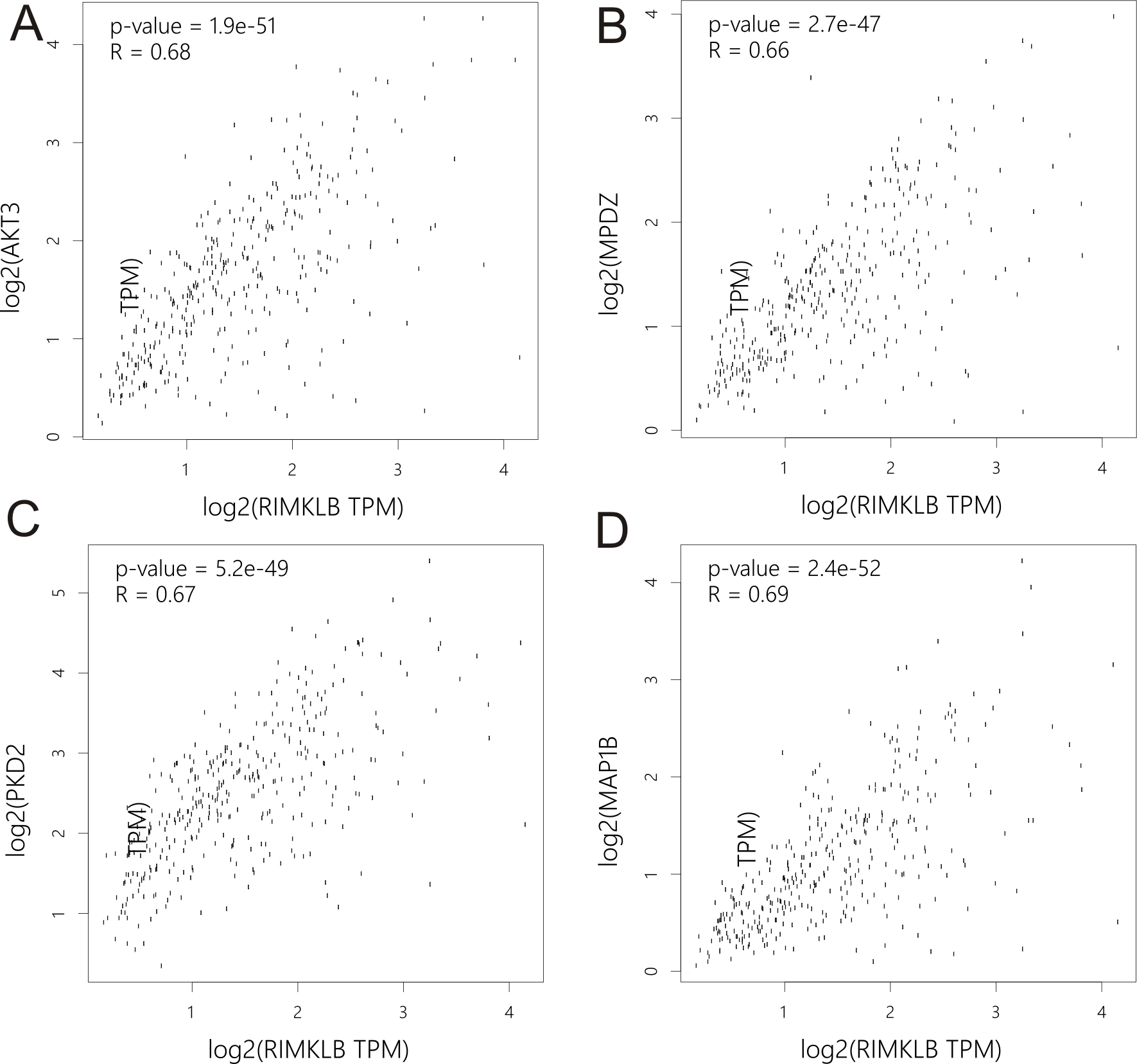

Supplement: Supplementary file 4 [file Image2.TIF]

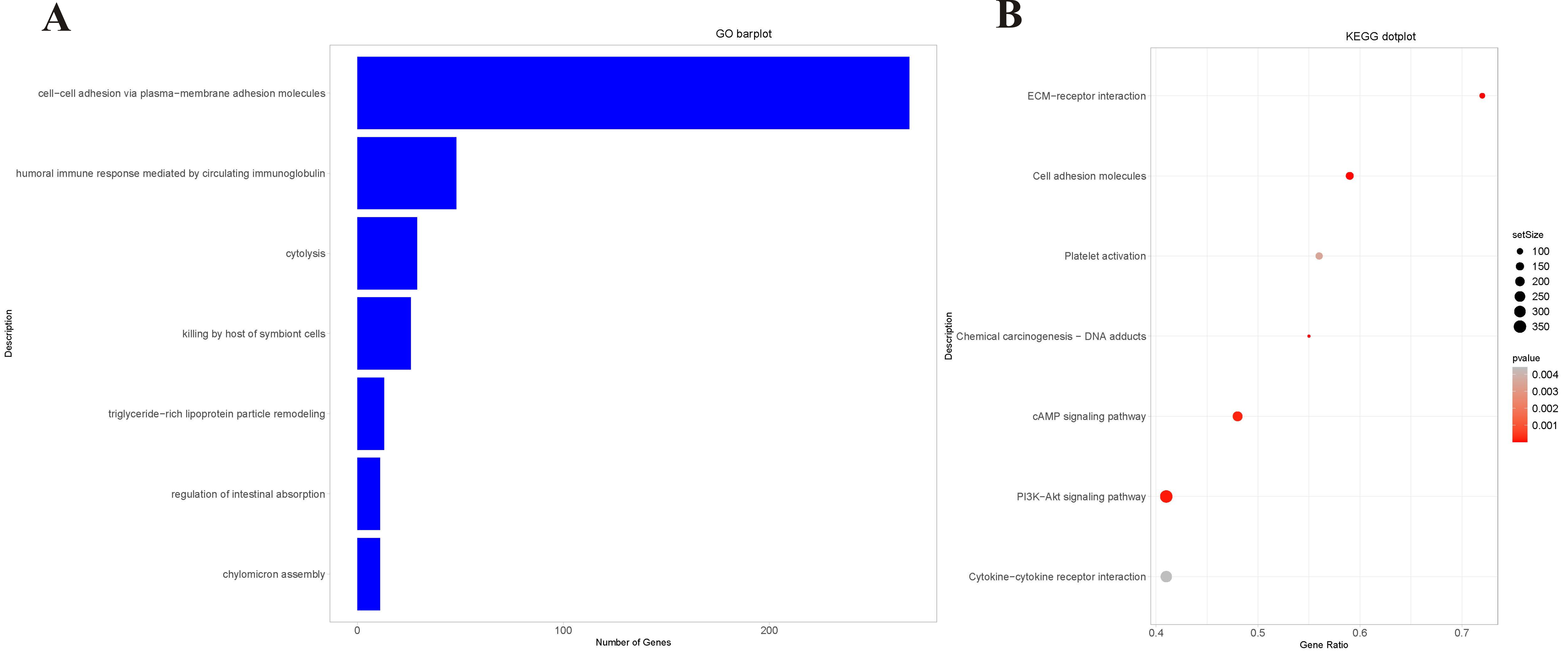

Supplement: Supplementary file 5 [file Image5.TIF]
